# Crystal structure and Hirshfeld surface analysis of 2-(4-nitro­phen­yl)-2-oxoethyl 2-chloro­benzoate

**DOI:** 10.1107/S2056989019014336

**Published:** 2019-10-31

**Authors:** S. N. Sheshadri, C. S. Chidan Kumar, S. Naveen, M. K. Veeraiah, Kakarla Raghava Reddy, Ismail Warad

**Affiliations:** a Department of Chemistry, GSSS Institute of Engineering and Technology for Women, Mysuru 570 016, Karnataka, India; b Department of Engineering Chemistry, Vidya Vikas Institute of, Engineering & Technology, Visvesvaraya Technological University, Alanahally, Mysuru 570 028, Karnataka, India; c Department of Physics, School of Engineering and Technology, Jain University, Bangalore 562 112, India; d Department of Chemistry, Sri Siddhartha Institute of Technology, Tumkur 572 105, Karnataka, India; e School of Chemical & Biomolecular Engineering, The University of Sydney, Sydney, NSW, Australia; f Department of Chemistry, Science College, An-Najah National University, PO Box 7, Nablus, West Bank, Palestinian Territories

**Keywords:** crystal structure, supra­molecular architecture, C—H⋯Cl hydrogen bonds, π–π inter­actions, Hirshfeld surface analysis

## Abstract

The title compound, C_15_H_10_ClNO_5_, is relatively planar with the two aromatic rings being inclined to each other by 3.56 (11)°. The central —C(=O)—C–O—C(=O)— bridge is slightly twisted, with a C—C—O—C torsion angle of 164.95 (16)°. In the crystal, mol­ecules are linked by C—H⋯O and C—H⋯Cl hydrogen bonds, forming layers parallel to the (101) plane. The layers are linked by a further C—H⋯O hydrogen bond, forming a three-dimensional supra­molecular structure. There are a number of offset π–π inter­actions present between the layers [inter­centroid distances vary from 3.8264 (15) to 3.9775 (14) Å]. Hirshfeld surface analyses, the *d*_norm_ surfaces, electrostatic potential and two-dimensional fingerprint plots were examined to verify the contributions of the different inter­molecular contacts within the supra­molecular structure. The shape-index surface shows that two sides of the mol­ecule are involved in the same contacts with neighbouring mol­ecules, and the curvedness plot shows flat surface patches that are characteristic of planar stacking.

## Abstract 2

The title compound, 2-(4-nitro­phen­yl)-2-oxoethyl 2-chloro­benzoate, is relatively planar with the two aromatic rings being inclined to each other by 3.56 (11)°.

## Chemical context   

Due to their numerous applications in various fields of chemistry, phenacyl benzoates are of great importance (Rather & Reid, 1919 ▸; Literák *et al.*, 2006 ▸; Sheehan & Umezawa, 1973 ▸; Huang *et al.*, 1996 ▸; Gandhi *et al.*, 1995 ▸; Zhang *et al.*, 2009 ▸). In continuation of our work on such mol­ecules (Kumar *et al.*, 2014 ▸; Chidan Kumar *et al.*, 2014 ▸), we report herein on the crystal and mol­ecular structures of 2-(4-nitro­phen­yl)-2-oxoethyl chloro­benzoate (I). Its crystal and mol­ecular structures are compared with those of 2-(4-nitro­phen­yl)-2-oxoethyl benzoate (II) (Sheshadri *et al.*, 2019 ▸), published by us recently, and further details of uses and applications of such mol­ecules are described therein.

## Structural commentary   

**Figure 1:**
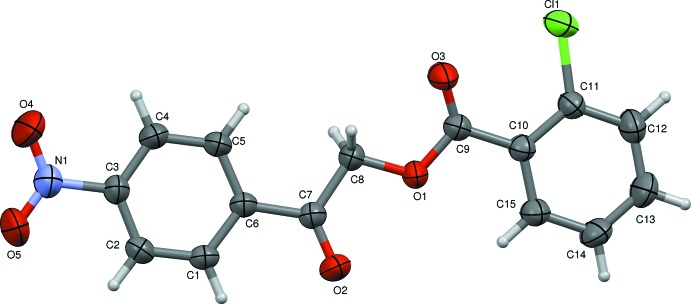
The mol­ecular structure of compound I, with the atom labelling. Displacement ellipsoids are drawn at the 50% probability level.

The mol­ecular structure of the title compound, I, is shown in Fig. 1 ▸. The compound is composed of two aromatic rings (C1–C6 and C10–C15) linked by the –C7(=O2)—C8—O1—C9(=O3)– bridge. The bond lengths and angles in I are normal and similar to those reported for compound II. The two benzene rings are inclined to each other by 3.56 (11)°, indicating that they are almost coplanar, as in the structure of II. The nitro group (N1/O4/O5) lies almost in the plane of the benzene ring (C1–C6), with a dihedral angle between the two planes of 5.4 (4)°; the torsion angles C4—C3—N1—O4 and C2—C3—N1—O5 are −5.4 (3) and −5.1 (4)°, respectively. Atom Cl1 is displaced by 0.0749 (8) Å from the plane of benzene ring C10–C15.
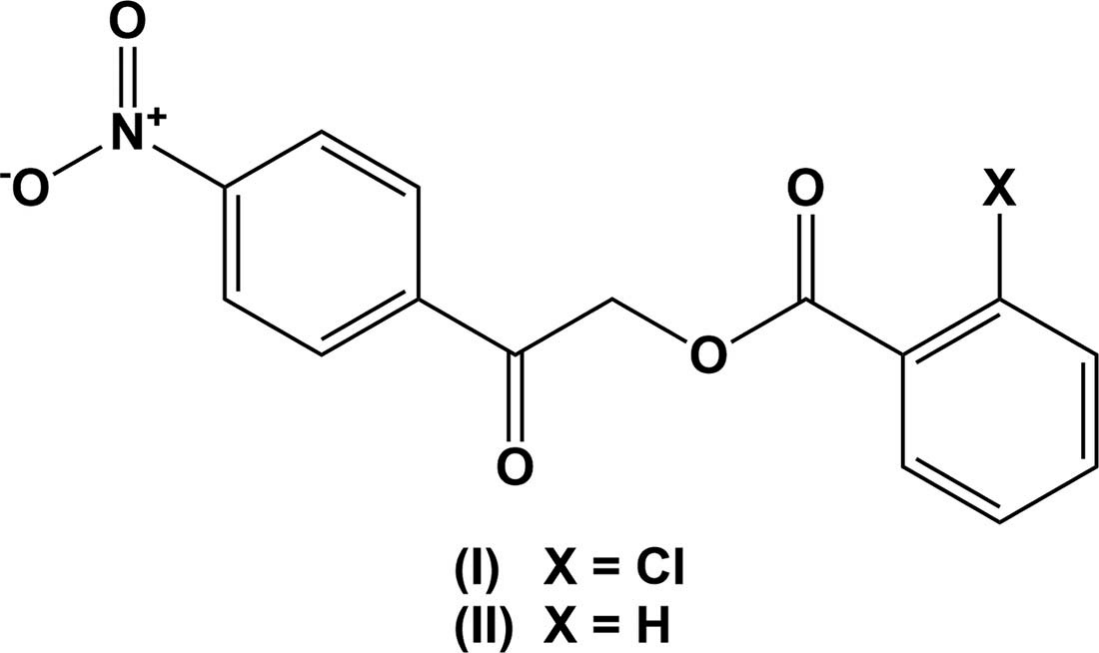


The overall mol­ecular conformation of I is characterized by three torsion angles, *viz.* τ_1_ (C11—C10—C9—O3), τ_2_ (C7—C8—O1—C9) and τ_3_ (O2—C7—C6—C1). Torsion angle τ_1_ at −12.5 (3)° signifies a certain noncoplanarity between the benzene ring (C10–C15) and the adjacent carbonyl group (C9=O3) as a result of steric repulsion between the substituent Cl1 and the adjacent carbonyl group C9=O3. This is also reflected in the torsion angle τ_2_ of −164.95 (16)°, between the two carbonyl groups, C7=O2 and C9=O3, which have a –*anti­periplanar* conformation. Torsion angle τ_3_, involving the benzene ring (C1–C6) and the adjacent carbonyl group (C7=O2), is −3.6 (3)° and indicates a –*synperiplanar* conformation. A comparison of the torsion angles in I and II, indicates that the insertion of the Cl atom in I has the most significant influence on torsion τ_2_, which is −164.95 (16)° in I compared to 174.08 (9)° in II. Torsion angles τ_1_ of −12.5 (3)° and τ_3_ of −3.6 (3)° are slightly larger than the values observed in II, *viz*. 9.60 (16) and 1.88 (15)°, respectively. Hence, compound I has a less planar conformation than unsubstituted compound II.

## Supra­molecular features   

**Figure 2:**
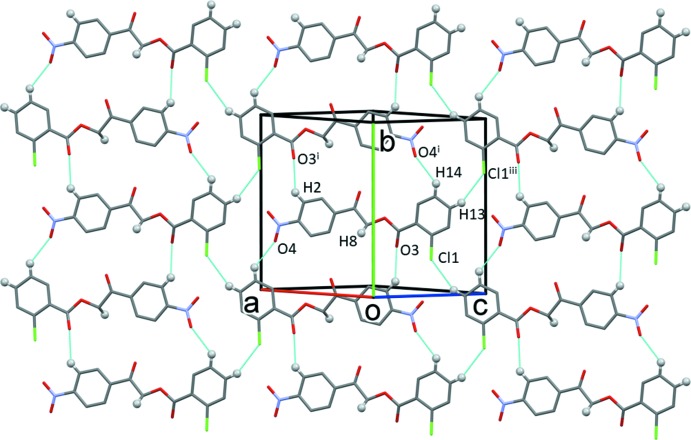
A view normal to the (101) plane of the crystal packing of compound I. The hydrogen bonds are shown as dashed lines (Table 1 ▸; symmetry codes as in Table 1 ▸), and, for clarity, only the H atoms involved in hydrogen bonding have been included.

**Figure 3:**
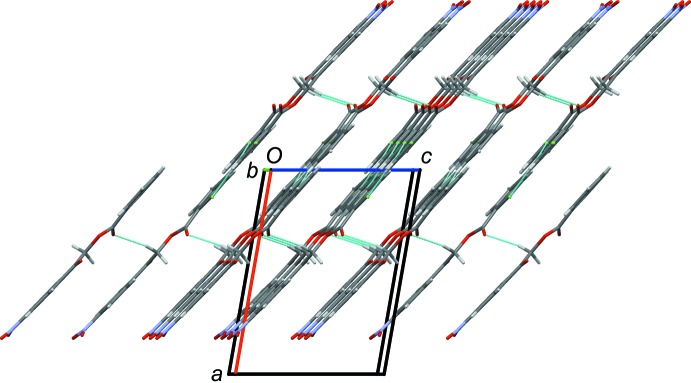
The crystal packing of compound I, viewed along the *b* axis, showing the layered stacking. For clarity, only the H atoms involved in hydrogen bonding have been included, and the hydrogen bonds are shown as dashed lines (Table 1 ▸).

The crystal structure of the title compound, is stabilized by inter­molecular hydrogen bonds of the types C—H⋯O and C—H⋯Cl (Table 1 ▸). Mol­ecules are linked by the C2—H2⋯O3^i^, C14—H14⋯O4^i^ and C13—H13⋯Cl1^iii^ hydrogen bonds to form layers lying parallel to the (101) plane; see Fig. 2 ▸ and Table 1 ▸. The layers are linked by C8—H8*A*⋯O3^ii^ hydrogen bonds and offset π–π inter­actions (see Table 2 ▸), forming a supra­molecular three-dimensional structure (Fig. 3 ▸).

## Hirshfeld surface analysis and two-dimensional fingerprint plots   

**Figure 4:**
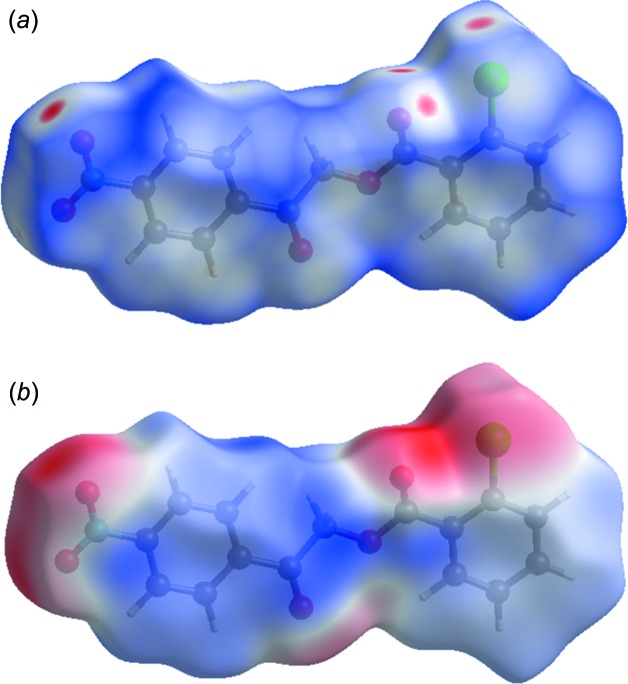
A view of the Hirshfeld surface of compound I, mapped over *d*_norm_.

**Figure 5:**
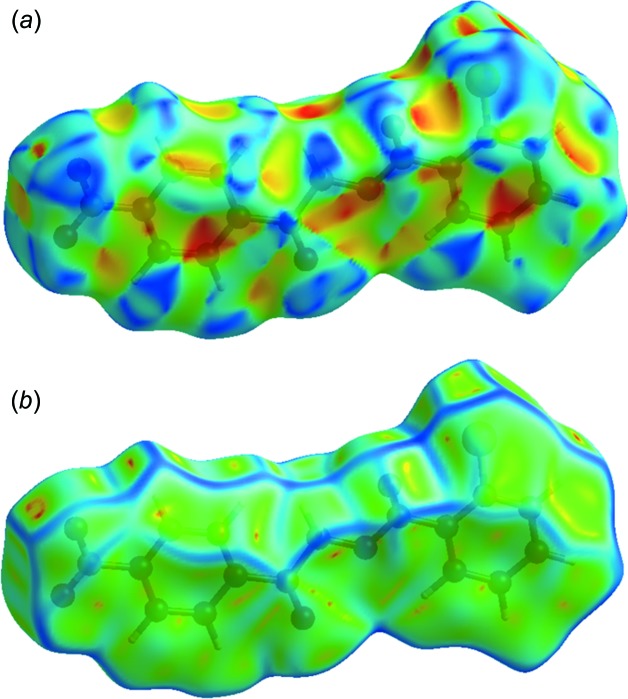
Hirshfeld surface of compound I, mapped over (*a*) the shape-index and (*b*) the curvedness.

The Hirshfeld surface analysis (Spackman & Jayatilaka, 2009 ▸) and the associated two-dimensional fingerprint plots (McKinnon *et al.*, 2007 ▸) were performed with *CrystalExplorer17* (Turner *et al.*, 2017 ▸). Hirshfeld surface analysis enables the visualization of inter­molecular inter­actions by different colours and colour intensity, representing short or long contacts and indicating the relative strength of the inter­actions. Fig. 4 ▸(*a*) shows the Hirshfeld surface mapped over *d*_norm_ (−0.154 to 1.305) and for Fig. 4 ▸(*b*) the electrostatic potential. The Hirshfeld surface illustrated in Fig. 4 ▸(*a*) reflects the involvement of different atoms with the inter­molecular inter­actions through the appearance of blue and red patches, which correspond to the regions of positive and negative electrostatic potential shown in Fig. 4 ▸(*b*). The shape-index surface (Fig. 5 ▸ *a*) clearly shows that the two sides of the mol­ecule are involved in contacts with neighbouring mol­ecules and the curvedness plot (Fig. 5 ▸ *b*) shows flat surface patches characteristic of planar stacking.

**Figure 6:**
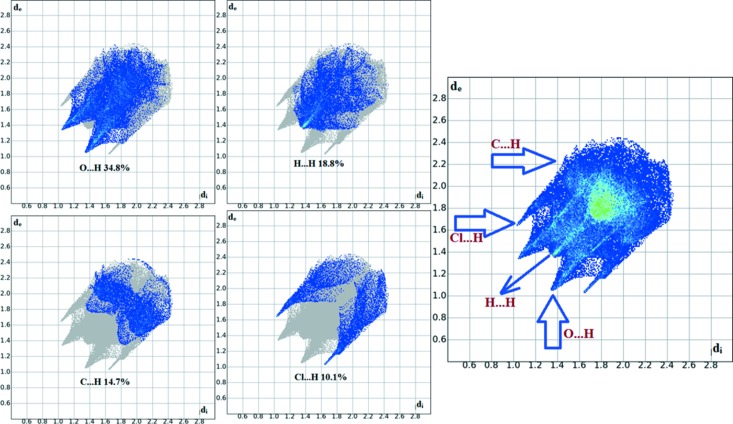
The two-dimensional fingerprint plots of compound I, showing the percentage contributions of all contacts and of individual atom–atom contacts.

The overall two-dimensional fingerprint plot for the title compound and those delineated into O⋯H/H⋯O, H⋯H, C⋯H/H⋯C and Cl⋯H/H⋯Cl contacts are illustrated in Fig. 6 ▸. The percentage contributions from the different inter­atomic contacts to the Hirshfeld surfaces are as follows: O⋯H (34.8%), H⋯H (18.8%), C⋯H (14.7%) and Cl⋯H (10.1%), shown in the two-dimensional fingerprint plots, respectively, in Fig. 6 ▸. The percentage contributions for other inter­molecular contacts are less than 5% in the Hirshfeld surface mapping.

## Database survey   

A search of the Cambridge Structural Database (CSD, Version 5.40, last update May 2019; Groom *et al.*, 2016 ▸) using 2-oxo-2-phenyl­ethyl benzoate as the main skeleton revealed the presence of 62 structures with different substituents on the terminal phenyl rings. In these structures, the two aromatic rings are inclined to each other by dihedral angles varying from *ca* 0 to 90°. There were seven structures with a nitro substituent on one of the aromatic rings. However, there is only one compound with the same skeleton as the title compound, *i.e.* 2-(biphenyl-4-yl)-2-oxoethyl 4-nitro­benzoate (CSD refcode CISSAB; Kwong *et al.*, 2017 ▸). Here the two aromatic rings are inclined to each other by *ca* 70.96°, compared to only 3.56 (11)° in the title compound. In the crystal structure of the recently published compound 2-(4-nitro­phen­yl)-2-oxoethyl benzoate (II) (Sheshadri *et al.*, 2019 ▸), this dihedral angle is 3.09 (5)°.

## Synthesis and crystallization   

The title compound, was synthesized as per the procedure reported earlier by Kumar *et al.* (2014 ▸). A mixture of 2-bromo-1-(4-nitro­phen­yl)ethanone (0.2 g, 0.5 mmol), potassium carbonate (0.087 g, 0.63 mmol) and 2-chloro­benzoic acid (0.156 g, 0.65 mmol) in di­methyl­formamide (5 ml) was stirred at room temperature for 2 h. After completion of the reaction, the reaction mixture was poured into ice-cold water. The solid product obtained was filtered, washed with water and recrystallized from ethanol to give colourless block-like crystals (m.p. 386–390 K).

## Refinement   

Crystal data, data collection and structure refinement details are summarized in Table 3 ▸. C-bound H atoms were positioned geometrically (C—H = 0.93–0.97 Å) and refined using a riding model, with *U*_iso_(H) = 1.2*U*_eq_(C).

## Supplementary Material

Crystal structure: contains datablock(s) global, I. DOI: 10.1107/S2056989019014336/su5521sup1.cif

Structure factors: contains datablock(s) I. DOI: 10.1107/S2056989019014336/su5521Isup2.hkl

CCDC references: 1449647, 1449647

Additional supporting information:  crystallographic information; 3D view; checkCIF report

## Figures and Tables

**Table 1:** Hydrogen-bond geometry (Å, °)

*D*—H⋯*A*	*D*—H	H⋯*A*	*D*⋯*A*	*D*—H⋯*A*
C2—H2⋯O3^i^	0.93	2.54	3.258 (2)	135
C8—H8*A*⋯O3^ii^	0.97	2.59	3.553 (3)	171
C13—H13⋯Cl1^iii^	0.93	2.82	3.670 (3)	153
C14—H14⋯O4^i^	0.93	2.50	3.211 (4)	134

Symmetry codes: (i) 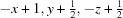; (ii) 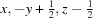; (iii) 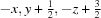.

**Table 2:** π–π contacts (Å, °) in the crystal of compound I *Cg*1 and *Cg*2 are the centroids of the C1–C6 and C10–C15 rings, respectively.

*Cg*(*I*)	*Cg*(*J*)	*Cg*(*I*)⋯*Cg*(*J*) (Å)	α (°)	β (°)	γ (°)	*CgI*_Perp (Å)	*CgJ*_Perp (Å)	Offset (Å)
*Cg*1	*Cg*1^iv^	3.9775 (14)	0.02 (10)	31.8	31.8	3.3791 (9)	3.3791 (9)	2.098
*Cg*1	*Cg*2^v^	3.8801 (14)	3.56 (11)	30.1	29.1	3.3895 (9)	3.3559 (10)	1.948
*Cg*2	*Cg*2^vi^	3.8264 (15)	0.00 (11)	24.8	24.8	3.4722 (10)	3.4722 (10)	1.608

Symmetry codes: (iv) −*x* + 1, −*y* + 1, −*z*; (v) −*x* + 1, −*y* + 1, −*z* + 1; (vi) −*x*, −*y* + 1, −*z* + 1.

**Table 3:** Experimental details

Crystal data	
Chemical formula	C_15_H_10_ClNO_5_
*M*_r_	319.69
Crystal system, space group	Monoclinic, *P*2_1_/*c*
Temperature (K)	294
*a*, *b*, *c* (Å)	12.6646 (18), 12.4099 (18), 9.0902 (13)
β (°)	99.947 (2)
*V* (Å^3^)	1407.2 (3)
*Z*	4
Radiation type	Mo *K*α
μ (mm^−1^)	0.30
Crystal size (mm)	0.55 × 0.26 × 0.19

Data collection	
Diffractometer	Bruker APEXII DUO CCD area-detector
Absorption correction	Multi-scan (*SADABS*; Bruker, 2012 ▸)
*T*_min_, *T*_max_	0.796, 0.946
No. of measured, independent and observed [*I* > 2σ(*I*)] reflections	36449, 4122, 2586
*R*_int_	0.058
(sin θ/λ)_max_ (Å^−1^)	0.705

Refinement	
*R*[*F* ^2^ > 2σ(*F* ^2^)], *wR*(*F* ^2^), *S*	0.054, 0.141, 1.06
No. of reflections	4122
No. of parameters	199
H-atom treatment	H-atom parameters constrained
Δρ_max_, Δρ_min_ (e Å^−3^)	0.26, −0.44

Computer programs: *APEX2* (Bruker, 2012 ▸), *SAINT* (Bruker, 2012 ▸), *SHELXS97* (Sheldrick, 2008 ▸), *Mercury* (Macrae *et al.*, 2008 ▸), *SHELXL97* (Sheldrick, 2008 ▸), *PLATON* (Spek, 2009 ▸) and *publCIF* (Westrip, 2010 ▸).

## supplementary crystallographic information

### Crystal data

**Table:**

C_15_H_10_ClNO_5_	*F*(000) = 656
*M**_r_* = 319.69	*D*_x_ = 1.509 Mg m^−^^3^
Monoclinic, *P*2_1_/*c*	Mo *K*α radiation, λ = 0.71073 Å
Hall symbol: -P 2ybc	Cell parameters from 6264 reflections
*a* = 12.6646 (18) Å	θ = 2.3–27.5°
*b* = 12.4099 (18) Å	µ = 0.30 mm^−^^1^
*c* = 9.0902 (13) Å	*T* = 294 K
β = 99.947 (2)°	Block, colourless
*V* = 1407.2 (3) Å^3^	0.55 × 0.26 × 0.19 mm
*Z* = 4	

### Data collection

**Table:**

Bruker APEXII DUO CCD area-detector diffractometer	4122 independent reflections
Radiation source: Rotating Anode	2586 reflections with *I* > 2σ(*I*)
Graphite monochromator	*R*_int_ = 0.058
Detector resolution: 18.4 pixels mm^-1^	θ_max_ = 30.1°, θ_min_ = 1.6°
φ and ω scans	*h* = −17→17
Absorption correction: multi-scan (SADABS; Bruker, 2012)	*k* = −17→17
*T*_min_ = 0.796, *T*_max_ = 0.946	*l* = −12→12
36449 measured reflections	

### Refinement

**Table:**

Refinement on *F*^2^	0 restraints
Least-squares matrix: full	Hydrogen site location: inferred from neighbouring sites
*R*[*F*^2^ > 2σ(*F*^2^)] = 0.054	H-atom parameters constrained
*wR*(*F*^2^) = 0.141	W = 1/[Σ^2^(*F*O^2^) + (0.0456*P*)^2^ + 0.8032*P*] WHERE *P* = (*F*O^2^ + 2*F*C^2^)/3
*S* = 1.06	(Δ/σ)_max_ < 0.001
4122 reflections	Δρ_max_ = 0.26 e Å^−^^3^
199 parameters	Δρ_min_ = −0.44 e Å^−^^3^

### Special details

**Table:**

Geometry. Bond distances, angles etc. have been calculated using the rounded fractional coordinates. All su's are estimated from the variances of the (full) variance-covariance matrix. The cell esds are taken into account in the estimation of distances, angles and torsion angles
Refinement. Refinement on F^2^ for ALL reflections except those flagged by the user for potential systematic errors. Weighted R-factors wR and all goodnesses of fit S are based on F^2^, conventional R-factors R are based on F, with F set to zero for negative F^2^. The observed criterion of F^2^ > 2sigma(F^2^) is used only for calculating -R-factor-obs etc. and is not relevant to the choice of reflections for refinement. R-factors based on F^2^ are statistically about twice as large as those based on F, and R-factors based on ALL data will be even larger.

### Fractional atomic coordinates and isotropic or equivalent isotropic displacement parameters (Å^2^)

**Table:**

	*x*	*y*	*z*	*U*_iso_*/*U*_eq_	
Cl1	0.13276 (6)	0.19871 (4)	0.64688 (9)	0.0704 (3)	
O1	0.32241 (12)	0.43016 (11)	0.42430 (17)	0.0451 (5)	
O2	0.44530 (13)	0.57459 (11)	0.32308 (19)	0.0553 (6)	
O3	0.31616 (15)	0.26717 (12)	0.5238 (2)	0.0662 (7)	
O4	0.8091 (3)	0.28826 (18)	−0.0648 (4)	0.1349 (15)	
O5	0.8233 (2)	0.45298 (17)	−0.1127 (3)	0.1031 (10)	
N1	0.78442 (18)	0.38048 (17)	−0.0532 (3)	0.0632 (8)	
C1	0.60280 (16)	0.53537 (15)	0.1467 (2)	0.0378 (6)	
C2	0.68013 (17)	0.51241 (15)	0.0615 (2)	0.0407 (6)	
C3	0.70239 (17)	0.40590 (16)	0.0379 (2)	0.0432 (7)	
C4	0.65143 (19)	0.32166 (16)	0.0961 (3)	0.0493 (7)	
C5	0.57408 (18)	0.34589 (15)	0.1802 (3)	0.0458 (7)	
C6	0.54870 (15)	0.45265 (14)	0.2066 (2)	0.0356 (6)	
C7	0.46407 (16)	0.48220 (15)	0.2957 (2)	0.0367 (6)	
C8	0.40417 (18)	0.38936 (16)	0.3492 (2)	0.0429 (6)	
C9	0.28099 (17)	0.35666 (15)	0.5069 (2)	0.0394 (6)	
C10	0.19286 (15)	0.40267 (15)	0.5758 (2)	0.0365 (6)	
C11	0.12673 (17)	0.33809 (16)	0.6478 (2)	0.0414 (6)	
C12	0.05046 (19)	0.3840 (2)	0.7205 (3)	0.0548 (8)	
C13	0.0388 (2)	0.4942 (2)	0.7222 (3)	0.0604 (9)	
C14	0.10067 (19)	0.55891 (18)	0.6499 (3)	0.0544 (8)	
C15	0.17715 (17)	0.51397 (16)	0.5769 (3)	0.0443 (7)	
H1	0.58650	0.60680	0.16450	0.0450*	
H2	0.71620	0.56730	0.02110	0.0490*	
H4	0.66890	0.25050	0.07900	0.0590*	
H5	0.53830	0.29040	0.21980	0.0550*	
H8A	0.37220	0.34550	0.26490	0.0510*	
H8B	0.45330	0.34460	0.41680	0.0510*	
H12	0.00710	0.34040	0.76810	0.0660*	
H13	−0.01150	0.52480	0.77300	0.0720*	
H14	0.09130	0.63320	0.64990	0.0650*	
H15	0.21870	0.55860	0.52770	0.0530*	

### Atomic displacement parameters (Å^2^)

**Table:**

	*U*^11^	*U*^22^	*U*^33^	*U*^12^	*U*^13^	*U*^23^
Cl1	0.0741 (4)	0.0363 (3)	0.1119 (6)	−0.0032 (3)	0.0471 (4)	0.0109 (3)
O1	0.0529 (9)	0.0353 (7)	0.0540 (9)	0.0031 (6)	0.0285 (7)	0.0035 (6)
O2	0.0648 (11)	0.0323 (7)	0.0754 (11)	−0.0003 (7)	0.0311 (9)	−0.0077 (7)
O3	0.0755 (12)	0.0382 (8)	0.0983 (14)	0.0158 (8)	0.0524 (11)	0.0190 (8)
O4	0.165 (3)	0.0587 (13)	0.222 (3)	0.0172 (14)	0.149 (3)	−0.0060 (16)
O5	0.1241 (19)	0.0699 (13)	0.144 (2)	−0.0058 (13)	0.1036 (18)	0.0043 (13)
N1	0.0670 (14)	0.0509 (11)	0.0825 (16)	−0.0031 (10)	0.0433 (12)	−0.0073 (11)
C1	0.0436 (11)	0.0268 (8)	0.0437 (11)	−0.0021 (7)	0.0095 (9)	−0.0019 (8)
C2	0.0446 (11)	0.0342 (9)	0.0456 (12)	−0.0058 (8)	0.0141 (9)	0.0031 (8)
C3	0.0444 (12)	0.0394 (10)	0.0504 (13)	−0.0019 (9)	0.0214 (10)	−0.0037 (9)
C4	0.0588 (14)	0.0296 (9)	0.0659 (15)	−0.0004 (9)	0.0284 (12)	−0.0048 (9)
C5	0.0554 (13)	0.0288 (9)	0.0591 (14)	−0.0051 (9)	0.0268 (11)	−0.0009 (9)
C6	0.0393 (10)	0.0295 (8)	0.0392 (11)	−0.0031 (7)	0.0106 (9)	−0.0026 (8)
C7	0.0412 (11)	0.0320 (9)	0.0383 (11)	−0.0015 (8)	0.0105 (9)	−0.0013 (8)
C8	0.0517 (12)	0.0341 (9)	0.0486 (12)	0.0005 (8)	0.0250 (10)	−0.0021 (8)
C9	0.0428 (11)	0.0322 (9)	0.0458 (12)	−0.0011 (8)	0.0149 (9)	0.0010 (8)
C10	0.0355 (10)	0.0339 (9)	0.0410 (11)	0.0012 (7)	0.0089 (9)	0.0032 (8)
C11	0.0409 (11)	0.0368 (9)	0.0491 (12)	−0.0001 (8)	0.0149 (9)	0.0046 (9)
C12	0.0500 (13)	0.0566 (13)	0.0644 (16)	0.0006 (11)	0.0288 (12)	0.0066 (12)
C13	0.0540 (14)	0.0560 (14)	0.0790 (18)	0.0104 (11)	0.0336 (14)	−0.0026 (13)
C14	0.0537 (14)	0.0369 (11)	0.0762 (17)	0.0096 (9)	0.0214 (13)	−0.0022 (10)
C15	0.0456 (12)	0.0343 (10)	0.0553 (13)	0.0018 (8)	0.0150 (10)	0.0030 (9)

### Geometric parameters (Å, º)

**Table:**

Cl1—C11	1.732 (2)	C10—C11	1.401 (3)
O1—C8	1.428 (3)	C10—C15	1.396 (3)
O1—C9	1.344 (2)	C11—C12	1.384 (3)
O2—C7	1.206 (2)	C12—C13	1.376 (4)
O3—C9	1.197 (2)	C13—C14	1.367 (4)
O4—N1	1.196 (3)	C14—C15	1.383 (3)
O5—N1	1.199 (3)	C1—H1	0.9300
N1—C3	1.470 (3)	C2—H2	0.9300
C1—C2	1.379 (3)	C4—H4	0.9300
C1—C6	1.396 (3)	C5—H5	0.9300
C2—C3	1.376 (3)	C8—H8A	0.9700
C3—C4	1.381 (3)	C8—H8B	0.9700
C4—C5	1.376 (3)	C12—H12	0.9300
C5—C6	1.394 (3)	C13—H13	0.9300
C6—C7	1.496 (3)	C14—H14	0.9300
C7—C8	1.506 (3)	C15—H15	0.9300
C9—C10	1.485 (3)		
			
C8—O1—C9	114.31 (15)	C10—C11—C12	120.71 (19)
O4—N1—O5	123.0 (3)	C11—C12—C13	120.0 (2)
O4—N1—C3	118.4 (3)	C12—C13—C14	120.5 (2)
O5—N1—C3	118.6 (2)	C13—C14—C15	120.1 (2)
C2—C1—C6	120.74 (17)	C10—C15—C14	121.0 (2)
C1—C2—C3	118.07 (18)	C2—C1—H1	120.00
N1—C3—C2	118.53 (18)	C6—C1—H1	120.00
N1—C3—C4	118.41 (19)	C1—C2—H2	121.00
C2—C3—C4	123.1 (2)	C3—C2—H2	121.00
C3—C4—C5	118.17 (19)	C3—C4—H4	121.00
C4—C5—C6	120.72 (19)	C5—C4—H4	121.00
C1—C6—C5	119.24 (18)	C4—C5—H5	120.00
C1—C6—C7	118.47 (16)	C6—C5—H5	120.00
C5—C6—C7	122.29 (18)	O1—C8—H8A	110.00
O2—C7—C6	122.01 (18)	O1—C8—H8B	110.00
O2—C7—C8	122.19 (19)	C7—C8—H8A	110.00
C6—C7—C8	115.80 (16)	C7—C8—H8B	110.00
O1—C8—C7	109.30 (16)	H8A—C8—H8B	108.00
O1—C9—O3	121.9 (2)	C11—C12—H12	120.00
O1—C9—C10	111.65 (16)	C13—C12—H12	120.00
O3—C9—C10	126.36 (19)	C12—C13—H13	120.00
C9—C10—C11	122.06 (17)	C14—C13—H13	120.00
C9—C10—C15	120.13 (18)	C13—C14—H14	120.00
C11—C10—C15	117.74 (18)	C15—C14—H14	120.00
Cl1—C11—C10	122.61 (16)	C10—C15—H15	120.00
Cl1—C11—C12	116.66 (17)	C14—C15—H15	119.00
			
C9—O1—C8—C7	−164.95 (16)	C5—C6—C7—O2	177.1 (2)
C8—O1—C9—O3	5.3 (3)	C5—C6—C7—C8	−2.8 (3)
C8—O1—C9—C10	−177.15 (15)	O2—C7—C8—O1	3.6 (3)
O4—N1—C3—C2	174.8 (3)	C6—C7—C8—O1	−176.59 (15)
O4—N1—C3—C4	−5.1 (4)	O1—C9—C10—C11	170.13 (17)
O5—N1—C3—C2	−5.4 (3)	O1—C9—C10—C15	−13.2 (3)
O5—N1—C3—C4	174.7 (3)	O3—C9—C10—C11	−12.5 (3)
C6—C1—C2—C3	−0.2 (3)	O3—C9—C10—C15	164.3 (2)
C2—C1—C6—C5	0.4 (3)	C9—C10—C11—Cl1	−6.6 (3)
C2—C1—C6—C7	−178.83 (17)	C9—C10—C11—C12	175.2 (2)
C1—C2—C3—N1	179.79 (19)	C15—C10—C11—Cl1	176.60 (17)
C1—C2—C3—C4	−0.4 (3)	C15—C10—C11—C12	−1.6 (3)
N1—C3—C4—C5	−179.5 (2)	C9—C10—C15—C14	−175.3 (2)
C2—C3—C4—C5	0.7 (4)	C11—C10—C15—C14	1.6 (3)
C3—C4—C5—C6	−0.5 (4)	Cl1—C11—C12—C13	−178.1 (2)
C4—C5—C6—C1	0.0 (3)	C10—C11—C12—C13	0.2 (3)
C4—C5—C6—C7	179.2 (2)	C11—C12—C13—C14	1.3 (4)
C1—C6—C7—O2	−3.7 (3)	C12—C13—C14—C15	−1.3 (4)
C1—C6—C7—C8	176.42 (17)	C13—C14—C15—C10	−0.2 (4)

### Hydrogen-bond geometry (Å, º)

**Table:**

*D*—H···*A*	*D*—H	H···*A*	*D*···*A*	*D*—H···*A*
C2—H2···O3^i^	0.93	2.54	3.258 (2)	135
C8—H8*A*···O3^ii^	0.97	2.59	3.553 (3)	171
C13—H13···Cl1^iii^	0.93	2.82	3.670 (3)	153
C14—H14···O4^i^	0.93	2.50	3.211 (4)	134

Symmetry codes: (i) −*x*+1, *y*+1/2, −*z*+1/2; (ii) *x*, −*y*+1/2, *z*−1/2; (iii) −*x*, *y*+1/2, −*z*+3/2.
